# Complementary medicine as a holistic approach for pain management in heart and lung transplantation

**DOI:** 10.1016/j.jhlto.2026.100593

**Published:** 2026-05-15

**Authors:** Jacinta Nalpon, Julien Fessler, Andrea Myers, Chinmay Patvardhan, Steven Tsui, Bastien Rodrigues

**Affiliations:** a Department of Anaesthesia and Intensive Care, Royal Papworth Hospital NHS Foundation Trust, Cambridge Biomedical Campus, Cambridge, England, UK; b Department of Anesthesiology, Hôpital Foch, Suresnes 92150, France; c Department of Anesthesiology, New York Presbyterian Hospital-Weill Cornell Medical Center, New York, NY; d Department of Anaesthesia and Intensive Care, Royal Papworth Hospital NHS Foundation Trust, Cambridge Biomedical Campus, Cambridge, England, UK; e Department of Cardiothoracic Surgery, Royal Papworth Hospital NHS Foundation Trust, Cambridge Biomedical Campus, Cambridge, England, UK; f Anesthesiology and Pain Medicine, University of Toronto, Toronto, Ontario, Canada; g Transitional Pain Service, Toronto General Hospital, Toronto, Ontario, Canada; h Department of Anesthesia and Pain Management, Toronto General Hospital, Toronto, Ontario, Canada

**Keywords:** heart and lung transplantation, pain, complementary medicine, mind-body technique, kinetic technique, quality of life

## Abstract

Perioperative pain management in thoracic organ transplantation continues to be challenging, with inadequate pain control correlating with extended recovery times, an increased incidence of chronic pain, and an overall poorer outcome. This review highlights the importance of a holistic approach in pain management following heart and lung transplantation, combining conventional pharmacological treatments with non-pharmacological techniques. The incidence of chronic pain in transplant recipients is high, with up to 75% of lung transplant recipients experiencing chronic pain within the first five years post-transplant. The contributing factors include surgical trauma, opioid induced hyperalgesia, graft-related discomfort, and musculoskeletal issues, whilst considering non-surgical influences such as biopsychosocial issues and gender. The implementation of complementary techniques for acute and chronic pain management includes mind-body and kinetic techniques. Recent research demonstrated the potential benefits of auricular acupuncture in reducing post-operative inflammation and pain. Early mobilization, specific respiratory exercises, and positive communication strategies are significant components of comprehensive pain management. Whilst evidence base for complementary interventions is still evolving, integrating them into post-operative care plans reflects a commitment to improving patient care.

Greater research should concentrate on evaluating the efficacy of complementary therapy in heart and lung transplant recipients to optimize pain management strategies and enhance post-transplant quality of life.

## Introduction

The nociceptive and neuropathic mechanisms associated with incision and intercostal nerve injury are common to all thoracic surgeries, with similar adverse consequences in terms of rehabilitation. However, unlike conventional thoracic surgery, patients undergoing lung transplantation are unique regarding preoperative frailty, anxiety upon the waiting transplant list, and the invasiveness of the surgery (bilateral thoracotomy). This explains why these patients' multidimensional aspects of pain management can be more complex. Thus, perioperative pain management in thoracic transplantations remains a significant challenge. Despite conventional medical therapies including opioids and regional anesthesia, post-operative pain can result in slower recovery and poorer outcomes. Inadequate pain control in the perioperative period is associated with the development of chronic pain. It has been established that chronic pain can result in the expression of molecular factors that are linked to the development of psychological syndromes such as depression and chronic anxiety.^1^ In these states, the neural loops in the brain that register pain signals are heightened and maintain constant vigilance for the onset of pain. This may explain why specific mind-body techniques can effectively treat persistent, unrelieved acute and chronic pain.

Perioperative pain management should start as soon as the patient is enrolled in a thoracic organ transplantation program. During this time, patients should be evaluated and given a comprehensive plan that includes conventional pharmacological treatments and incorporates non-pharmacological approaches for optimal outcomes. This preemptive combined strategy can lead to a more favorable response. Addressing pain holistically encourages the practitioner to explore additional interventions to alleviate pain and enhance the overall pain experience.

An innovative view would involve caring for physical, psychological, and emotional pain through a multidisciplinary team approach. Patients are increasingly seeking alternative and complementary medicine for post-surgical pain relief.

Azem K. et al^2^ provides an overview of the importance of regional anesthesia targeting different incisions in lung transplantation. This article reviews complementary methods to provide a more holistic approach to pain management after heart and lung transplantation.

## Definition of post-surgical pain

Acute post–surgical pain results directly from surgical trauma. Its severity depends on the type of incision, the extent of surgery, the presence of chest drains, and the degree of tissue damage or injury. The response to pain is complex. Pain intensity is influenced not only by the surgical insult but also by physiological and psychological factors. In the physiology of pain, four components must be assessed: sensory-discriminative, affective, and emotional response, cognitive process, and behavioral response. The International Association for the Study of Pain (IASP) defines chronic postsurgical pain as pain that develops or increases in intensity after a surgical procedure or a tissue injury and persists beyond the healing process for at least three months after the surgery or tissue trauma.^3^ Thoracic and cardiac surgeries are major procedures that often result in significant postoperative pain due to large incisions, rib retraction, prolonged chest drains, and immobility.

### Prevalence of chronic pain in transplant recipients

Over recent years, the survival rate post-lung transplant has improved significantly, exceeding 70% at 5 years in some studies.^4^ However, improvements in quality of life have only recently been evaluated. An important determinant of quality of life for these patients is their ability to live without chronic pain. The intention is to prevent and reduce acute pain, which in turn can reduce the incidence of chronic pain with its related complications/outcomes. Severe alteration of quality of life has been reported in patients awaiting lung transplantation.^5^ In a French high-volume center, 59% of patients reported preoperative pain, and 36% reported significant anxiety while on the waiting list.^6^ Chronic postoperative pain was widespread in the first five years after a lung transplant, affecting 54.5–75% of recipients. Chronic pain is also commonly reported in patients following heart transplantation.^7^

### Clinical importance of chronic pain in thoracic transplant patients

#### Factors associated with postoperative pain after lung transplantation

**Figure 1:**
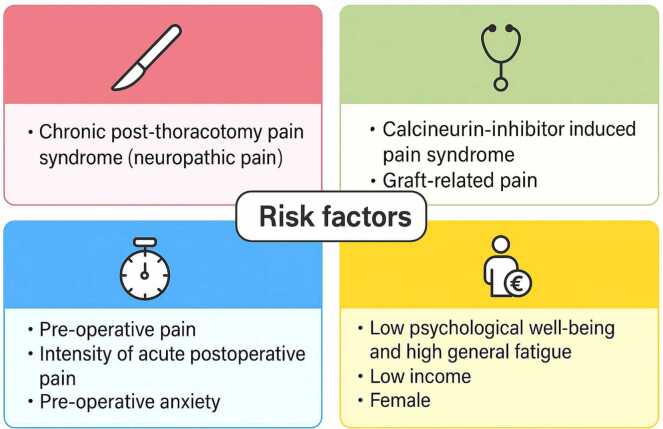
Post transplant patient related pain syndromes.

Different types of pain can occur due to various syndromes (Figure 1). In lung transplantation, chronic post-thoracotomy pain syndrome (CPTPS) is a frequent and clinically significant postoperative complication.^8^ Although neuropathic mechanisms related to intercostal nerve injury are common and may manifest as burning, stabbing, or tingling sensations, the pain syndrome is often multifactorial and not exclusively neuropathic in nature. For heart transplantation, post-sternotomy pain syndrome is persistent pain from sternum division and wire fixation, which is also often neuropathic.^9^ Also, other types of transplant-related pain exist. Calcineurin-inhibitor-induced pain syndrome is a medication-induced pain or neuropathy of the bones or joints, related to immunosuppressants.^10^ Graft-related pain is pain associated with the transplanted organ, due to inflammation of the lung allograft, pleural or pericardial irritation, or chronic rejection. Musculoskeletal pain is due to prolonged immobility, deconditioning, or postural imbalances (poor posture, scapular dyskinesis, and rib stiffness due to prolonged immobilization).

Alongside the known surgical risk factors for chronic postoperative pain, there are numerous non-surgical risk factors, including the presence of pre-operative pain, the intensity of acute postoperative pain, anxiety, low psychological well-being, high general fatigue, low income, and gender (women reported significantly worse pain intensity than men in a more affective and sensory dimension of pain).^11^, ^12^

### Clinical consequences

In the pre-operative period, pain may limit engagement with rehabilitation, affecting the ability of lung transplant recipients to perform deep breathing exercises, thus increasing the risk of atelectasis and pneumonia, impaired cardiac rehabilitation participation (pain discourages physical activity increasing the risk of deconditioning and cardiovascular complications); decreased physical activity and deconditioning (muscle weakness and postural imbalances worsen pain over time), and higher risk of opioid misuse (lung transplant recipients often require careful opioid management due to the risk of respiratory depression). Although the Pain Intensity Score (PIS), which is related to general fatigue, physical fatigue, and reduced activity, is associated with chronic pain,^9^ effective pain management is essential for preventing complications, enhancing recovery, and improving long-term outcomes. Even patients with mild pain reported a worse quality of life than the general population, and are less likely to return to a professional activity.^7^ Supplementing conventional pain management strategies with complementary therapies can enhance pain control and promote lung function, mobility, and overall well-being.

### Complementary therapies

**Figure 2:**
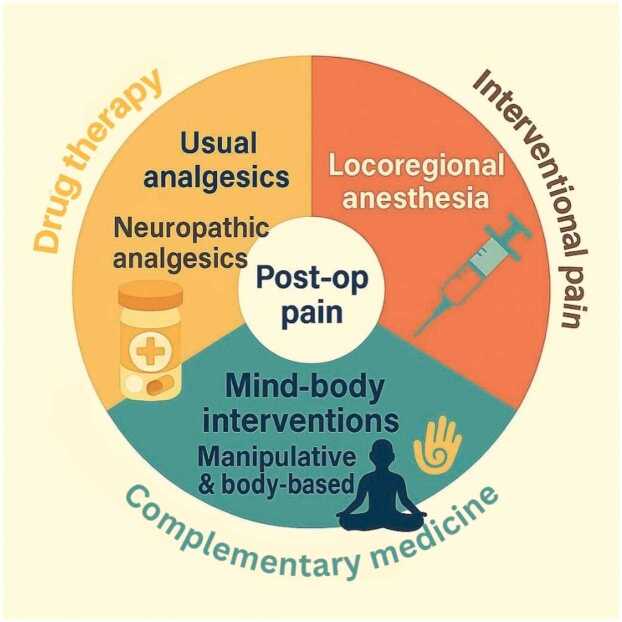
Patient centered approach to pain management.

A patient-centered approach to pain treatment, in addition to medication and regional analgesia, may lead to better outcomes and patient satisfaction (Figure 2). Complementary approaches to acute and chronic pain are commonly divided into mind–body interventions (e.g., muscle relaxation, hypnosis, guided imagery, yoga, and biofeedback)^13^ and somatic or kinetic techniques such as transcutaneous electrical nerve stimulation (TENS) and physiotherapy. Although acupuncture and acupressure are sometimes grouped with mind–body therapies, they are more commonly classified within traditional Chinese medicine, whereas auricular acupuncture (auriculotherapy) is generally considered a form of reflexotherapy.

Non-pharmacological interventions focus on psychological therapies, devices, e.g., transcutaneous electrical nerve stimulation (TENS), and physiotherapy, whilst complementary medicine/therapies can overlap with conventional medical treatments, e.g., Mindfulness, acupuncture/acupressure, and massage. Non-pharmacological interventions and complementary medicine/therapies require more robust, research-based evidence to substantiate their effectiveness. Together, they provide a holistic approach to transplant patient care.

### Mindbody techniques

Multiple studies have provided evidence of disparities in pain perception among different ethnic groups.^14^ Cultural factors are relevant to the expression of pain, coping mechanisms, communication, religious beliefs, and values. It also varies with age and the experience of pain. Thus, strategies for pain management should incorporate cultural awareness among healthcare practitioners, the design and implementation of culturally appropriate pain assessment tools, and encourage patients to discuss their pain experiences and cultural beliefs and values during pre-operative assessment clinics.

The quality of evidence supporting the efficacy of mind-body techniques in improving post-surgical outcomes remains limited.^15^ Research interest in the application of these techniques after solid organ transplantation has only begun to be evaluated.^16^ Auriculotherapy was first described in 1957 by Paul Nogier as a reflex-based approach founded on the concept of the auricular homunculus—a somatotopic representation of the body mapped onto specific points of the ear.^16^ While some authors include auriculotherapy within the broader framework of acupuncture and traditional Chinese medicine, many consider it a distinct modality. Contemporary neurophysiological understanding suggests that stimulation of the auricle modulates autonomic and central nervous system pathways via its rich innervation by cranial and spinal nerves, including the vagus, trigeminal, and great auricular nerves. This neuroanatomical configuration facilitates direct communication with the central nervous system, influencing key pain-processing areas and homeostatic regulation. Stimulation of these auricular points has been shown to modulate the release of neurotransmitters, including serotonin, dopamine, and norepinephrine, which play crucial roles in pain modulation and emotional regulation.^17^ Auricular acupuncture is considered a safe intervention with a favorable risk profile compared to many standard pharmacological treatments. Its combination of non-invasiveness and cost-effectiveness positions regular acupuncture as a valuable complementary or alternative approach in comprehensive pain management strategies.^18^, ^19^ Whilst the evidence base for some of these interventions continues to evolve, the integration into post-operative care protocols reflects a commitment to improving patient outcomes through comprehensive non-pharmacological approaches and to avoid the use of opioids for extended periods.

Recently, a study of patients undergoing knee surgery showed that auricular acupuncture reduced serum C-reactive protein levels 1 day after surgery, indicating a significant anti-inflammatory effect. This finding aligns with previous research showing that acupuncture, especially at specific points, can modulate inflammatory responses by decreasing pro-inflammatory cytokines and enhancing anti-inflammatory mechanisms.^20^ Large cohort data have indicated that body acupuncture is safe in patients taking newer oral anticoagulants, with only minor, self-limiting microbleeding with no major bleeding reported.^21^, ^22^

There are currently no specific safety studies of auriculotherapy in patients on novel oral anticoagulants (NOACs) or direct oral anticoagulants (DOACs). As Auricular acupressure with seeds do not pierce the skin, the risk of bleeding is minimized. Current practice at Papworth/Cambridge Transplant Center in the UK allows the auricular acupressure seeds to remain in-situ for 6 days, providing ongoing non-pharmacological support. Auricular acupressure also encourages the release of endorphins, reports of better anxiety management, improvement in activity focus, better quality of sleep and significant reduction of pain. Reliance on pharmacological analgesia is therefore minimized.

The combined effect of these interventions - effective pain management, reduced opioid reliance, improved rehabilitation techniques, and inflammation control - contributes to greater efficiency in healing and recovery outcomes. This comprehensive approach addresses immediate postoperative concerns and fosters long-term patient well-being, potentially reducing the incidence of chronic pain and improving overall quality of life following heart and lung transplantation.

Similarly, hypnosis is a complementary tool that has shown its usefulness in perioperative medicine, and especially for pain. It is called hypnoanalgesia. It can be delivered as a formal hypnosis beginning with a formal hypnotic induction, or as a conversational hypnosis using verbal suggestions.^23^ It has also been proposed that hypnoanalgesia can be delivered using new technology such as virtual reality. The three-dimensional visual and auditory immersion encourages the patient to dive into the induction independently. In a single-center randomized controlled trial, Malard et al. reported a reduction of procedure-related parasympathetic tone as measured by a nociceptive monitoring (analgesia nociception index) during oocyte retrieval performed under virtual reality hypnosis associated with local anesthesia, compared to local anesthesia alone.^24^ Interestingly, it may be possible to teach patients to self-administer hypnosis for pain relief. Michel-Cherqui et al. randomized 78 patients to study the efficacy of teaching self-hypnosis at the enrollment of a lung transplantation program to improve patients’ pain and quality of life after lung transplantation. The practice of self-hypnosis was high before transplantation (76.6%) but reduced to 32% in the intensive care unit and increased up to 52% 3 months after surgery. Unfortunately, the differences between groups did not reach statistical significance (p=0.07).^25^ Although the main result of this study was negative, the authors provided important insights into its limitations, notably the time delay between the initial teaching session and the eventual surgery, and the type of support available to reinforce and refresh the acquisition of self-hypnosis. Other limiting factors for the success of self-hypnosis may include the health care provider's confidence in the effectiveness of this approach, as well as the patient's motivation and cooperation.

During preoperative clinic consultation, information-giving before surgery prepares the patient and their carer(s) for what is to be expected with discussions addressing the anesthesia, possible surgical incisions, post-operative pain, available therapies, and potential repercussions. It is essential for patients to be informed before a procedure to allay anxiety and stress. However, because patients tend to focus on the details of the transplant procedure and the prospect that they may finally be able to function normally again after the transplant, they may only take in a limited amount of additional information or nothing at all. Care must be taken when choosing the words being used; if wisely used, they can be a powerful ally.^26^, ^27^ Incorrectly selected, they may induce a nocebo effect on pain sensation and cause more anxiety, as shown in neurobiological mechanisms and functional magnetic resonance imaging.^26^, ^28^, ^29^ Positive communication and benevolence can strengthen the rapport between the patient and the healthcare professional. This can help the patient to accept their new health status and lifestyle and enhance their coping mechanisms. It can also help them to maintain their mental well-being, reducing the risks of depression and post-traumatic stress. Specific strategies can be used to achieve these ends such as active listening (allowing patients to express concerns without interruption and acknowledge their emotions), clear and compassionate communication (using simple, non-technical language to check for understanding), empathy and encouragement (showing genuine care by reassuring patients about their progress and celebrating small victories), patient-centered approach (involving patients in decision-making, respecting their preferences and concerns), and holistic care (addressing not just medical needs but also psychological and social challenges through multidisciplinary support).

Martin AK et al.^30^ proposed a multidisciplinary vision of the perioperative management of lung transplantation as if it were the patient (*How we would treat our own lung transplantation*). They suggested letting the patient choose the music during their anesthetic induction as musicotherapy to reduce anxiety.

### Kinetic techniques

Early mobilization and targeted respiratory exercises can significantly improve rehabilitation outcomes. Encouraging patients to engage in deep breathing exercises and teaching effective coughing techniques can improve lung function, prevent atelectasis, and facilitate sputum clearance. When combined with proper pain management, these interventions enable patients to participate fully and at a more advanced level in the recovery process, leading to improved, more favorable overall outcomes.

The Foch Lung transplant team recently studied^31^ the implementation of a “toolbox” of complementary techniques before lung transplantation during an evaluation period before enrollment on the waiting list. Patients were offered at least one training session on different approaches such as relaxation, self-hypnosis, sophrology, and holistic gymnastics, and they were encouraged to practice them regularly. Almost 60% were evaluated at the fourth postoperative month, representing more than 4000 cumulative sessions. Relaxation was frequently used before lung transplantation and had a good self-appropriation. After transplantation, relaxation and transcutaneous electrical nerve stimulation (TENS) were the most commonly used techniques, with TENS showing better autonomy, usability, adaptation, and compliance. In a secondary analysis, this study found the percentage of patients with pain close to the proportion at inclusion but with a significant increase in neuropathic pain patients (3.4% at inclusion and 27.6% at three months; *p* = 0.0008), which is consistent with data in the literature suggesting an increase in neuropathic pain following thoracic surgery. A global improvement in quality of life was also reported in this secondary analysis, with anxiety and depression significantly reduced, suggesting a positive impact of these complementary therapies.

Other kinetic techniques are also available. Heat pads, massage, drain taping, and the application of sternal bands are integrated with standard pain management treatment after lung transplantation at the Papworth/Cambridge Transplant Center in the UK. However, no formal evaluation of these interventions has been carried out yet.

To our knowledge, there has been no study on the efficacy of complementary therapies for postoperative pain following heart transplantation. Because the risk factors and prevalence of pain appear to be similar in patients undergoing lung transplantation, complementary therapies may also have a useful role following heart transplantation. From discussions with colleagues at different centers, these methods are more widely used than the literature suggests, both informally and institutionally. Their use should be promoted because of their low cost, ease of use, and the very low incidence of undesirable effects.

## Conclusion

The management of chronic pain in lung and heart transplant recipients remains a challenge due to the complexities of surgery, immunosuppressive therapy, and long-term rehabilitation. While pharmacological analgesics and regional anesthesia play a significant role, complementary therapies such as mind-body interventions and physical therapies can offer valuable adjuncts to conventional pain management options. These practices will come with cultural change from each stakeholder in the approach to patients towards thoracic transplantation. Combining these therapies with physical rehabilitation and psychosocial support from the multidisciplinary team for assessment and management of pain, where needed, is essential to optimizing long-term outcomes for transplant recipients experiencing chronic pain. Further research is needed to establish standardized protocols and assess the long-term efficacy of these interventions in transplant populations. Finally, while evidence is still emerging, the integration of complementary therapies and positive communication strategies into post-transplant care shows potential for improving pain management, reducing opioid dependence, and enhancing overall quality of life for heart and lung transplant recipients.

## Contribution

All authors contribute equally to writing, review, and editing

## Declaration of Competing Interest

None in relation to the present work

The author is a Guest Editor for this journal and was not involved in the editorial review or the decision to publish this article
